# A Book Not for Sale

**Published:** 1860-12

**Authors:** 


### A BOOK NOT FOR SALE.

We have just published a new book, of 300 pages, twelvemo, entitled, Sleep. The preface reads thus:

"It is the end and aim of this book to show that as a means of high health, pure blood and a strong mind to old and young, sick or well, each one should have a single bed, in a large, clean, light room, so as to pass all the hours of sleep in a pure fresh air, and that those who fail in this, will in the end fail in health and strength of limb and brain, and will die while yet their days are not all told."

The first chapter is on "Sleeping with the Old," and begins as follows:

"On a beautiful September morning, in the year eighteen hundred and fifty-nine, a note was found on the author's table, in a handwriting which was immediately recognized as that of a wife and mother of high culture, in behalf of a young sister, whom she had hoped would have grown up as healthful, as beautiful, and as accomplished as herself; but the lovely blossom seemed to be fading in its unfolding, and the communication was a history of the case intended to give the physician an idea of its nature and its needs."

Next follows one of the sweetest descriptions of a human cherub and its fading, failing, falling into the cold, dark grave so soon! The idea of the book is that, as we spend a clean third of our existence in our chambers, it is absolutely essential to sound health and vigor of constitution, that a pure air be breathed all that time. The sources of impurity are enumerated. The effects of breathing these impurities are described in all degrees, from the slow poisoning of months and weary years, without the victims being conscious of the fact or the cause, to the instantaneous death. The methods of preventing these impurities are clearly described, with the great wrong done to ourselves, and the inhumanity to our children, by their neglect. It is argued that the young should not sleep with the old, the well with the sick, the strong with the feeble, as pernicious results follow to one party, without any possible good to the other. Ill effects follow from children sleeping with one another, bad habits are formed, with the horrible results of their indulgence; the impositions practiced on the suffering by unprincipled individuals and societies with benevolent names; the only safe and efficient hygienical means are pointed out, and which should be under parental application, the intervention of third parties being unnecessary. In describing the manner in which chambers may be supplied with a pure air, the subject of building, warming, and ventilating houses is discussed, and the latest plans described. Striking authenticated facts are presented, which show how bodily emanations speedily corrupt the air of a whole room, and how they may destroy fifty or a hundred lives in a few hours. The remedy proposed is large rooms and separate beds for all. The book contains information which ought to be possessed by every family, by every parent who has a child yet to reach twenty-one years of age, and by every human being who sleeps within any four walls. Supposing that about a third of our subscribers might want such a book, we have printed an edition of that proportion; it will be furnished in the order of application; the dilatory will have to go without one. As a means of making a small edition save us from actual loss, the following offer is made to those who desire to possess it. Three dollars will pay for the book and two subscriptions to the JOURNAL OF HEALTH for one year; or for the book and the JOURNAL OF HEALTH with the FIRESIDE MONTHLY for one year, the latter being separately $1.50 a year. The object of this offer is to induce our subscribers to add to our subscription-list, by using their influence in inducing some friend to take our MONTHLY; and for the time and trouble expended in using such influence, they will be paid by getting a book which, singly, money will not at present buy. Any one sending three subscriptions to the JOURNAL OF HEALTH, or two to the FIRESIDE, will be entitled to the book for their trouble.

